# The predictive value of Cardiodynamicsgram in myocardial perfusion abnormalities

**DOI:** 10.1371/journal.pone.0208859

**Published:** 2018-12-17

**Authors:** Xunde Dong, Jinhe Zhang, Hongji Lai, Min Tang, Shanxing Ou, Jianhong Dou, Cong Wang

**Affiliations:** 1 School of Automation Science and Engineering, South China University of Technology, Guangzhou, Guangdong, China; 2 Department of nuclear medicine, Guangzhou general hospital of PLA, Guangzhou, Guangdong, China; 3 Department of Cardiology, State Key Laboratory of Cardiovascular Disease, Cardiovascular Institute, Fuwai Hospital, National Center for Cardiovascular Diseases, Chinese Academy of Medical Sciences, and Peking Union Medical College, Beijing, China; 4 Department of radiology, Guangzhou general hospital of PLA, Guangzhou, Guangdong, China; 5 Department of anesthesiology, Guangzhou general hospital of PLA, Guangzhou, Guangdong, China; Ziekenhuisgroep Twente, NETHERLANDS

## Abstract

Myocardial perfusion abnormalities are the first sign of the ischemic cascade in the development of coronary artery disease (CAD). Thus, the early detection of myocardial perfusion abnormalities is significant for the prevention of CAD. Recently, a novel noninvasive method named Cardiodynamicsgram (CDG) has been proposed for early detection of CAD. This study aims to evaluate the predictive value of CDG in myocardial perfusion abnormalities for suspected ischemic heart disease. In the study, 86 suspected patients were enrolled. Standard 12-lead ECG and CDG were performed simultaneously before single-photon emission computed tomography (SPECT) myocardial perfusion imaging (MPI). Diagnostic accuracy of CDG for myocardial perfusion abnormalities detection is assessed using SPECT MPI as the reference standard. Of these 86 suspected patients, 37 patients were positive in CDG, 49 patients were negative in CDG. Diagnostic accuracy of CDG at presentation for myocardial perfusion abnormalities was 84.9%, sensitivity 84.0%, and specificity 89.4%. Furthermore, of the 10 patients whose SPECT MPI results are reverse redistribution, 9 patients were positive in CDG. Underlying causes of false positive CDG findings included the factors that can change the stability of cardiac electrical conduction and measurement noise. Myocardial remodeling in patients with old myocardial infarction might be the major cause of false negative findings. Results show a good consistency between the CDG and SPECT MPI in evaluating myocardial perfusion abnormalities. It suggests that CDG might be used as a cost-effective tool for assessing the myocardial perfusion abnormalities in the clinic.

## Introduction

At present, more than 7 million people die from coronary artery disease (CAD) in the world every year, and the number is expected to increase by more than three times to 23.3 million by 2030 [1]. In the development of CAD, the first sign of the ischemic cascade is myocardial perfusion abnormalities. Thus, early detection of myocardial perfusion abnormalities is important to reduce the morbidity and mortality of CAD [2]. Single photon-emission computed tomography (SPECT) is the most common noninvasive method used to evaluate myocardial perfusion [3–5]. In the USA alone, there are about 5 million inspections of SPECT were carried out every year [6]. The result of SPECT is one of the most important basis for the decision of revascularization [2]. Compared with other invasive detection methods (e.g., coronary angiography), SPECT has remarkable advantages of being cost-effective and risk free.

Recently, a novel noninvasive method named Cardiodynamicsgram (CDG) has emerged as a complementary diagnostic tool to conventional electrocardiograms (ECG) [7], which aimed to improve the detection accuracy of ischemia diseases. It is a 3D visualization of the cardiodynamics information extracted from the ST-T segments of standard 12-lead ECG by using deterministic learning theory. It is found there exist significant correlations between CDG morphology and ischemia, which can be used for ischemia detection. To improve the clinical utility, the CDG morphology was characterized quantitatively, and a diagnostic criteria for ischemia detection was proposed in [8]. It is as simple as routine ECG, but its sensitivity and specificity are higher than both routine 12-lead ECG and exercise ECG. Several articles have reported and shown the value of CDG for CAD early diagnosis [7–10]. This study sought to further evaluate the clinical utility of CDG in predicting the myocardial perfusion abnormalities for suspected ischemic heart disease using SPECT MPI as the reference standard.

## Materials and methods

### Patients

The study was performed at Guangzhou general hospital of PLA, China from May 2014 to November 2014. Ethical approval for the study was obtained from the Ethics Committee of Guangzhou general hospital of PLA. The Ethics Committee approved a verbal consent procedure as sufficient because the study was signal-based, and did not include any human specimens. Inclusion criteria were suspected patients with myocardial ischemia for SPECT MPI, and had symptoms of myocardial ischemia (e.g., chest pain). The suspicion of myocardial ischemia was based on the clinical assessment, including cardiac symptoms, risk factors, physical examination, blood analysis, and exercise ECG test. All enrolled patients received a CDG examination within the same day before the SPECT MPI. A total of 88 patients were evaluated observing the inclusion criteria, and informed consent was obtained. However, 2 patients were excluded as a result of incomplete clinical data. Therefore, the total population comprised 86 patients, 61 men and 25 women. Patient characteristics are presented in Table 1.

**Table 1:** The characteristics of the 86 patients.

Characteristic	
Ages(years)	52.5±32.5
Female	25(29.1%)
Chest pain	70(81.4%)
Ejection fraction(%)	56±34
Hypertension^†^	37(43.0%)
Diabetes mellitus	25(29.1%)
Dyslipidemia^‡^	32(37.2%)

^†^Hypertension was defined as a mean systolic blood pressure 140 mmHg, diastolic blood pressure 90 mm Hg, and/or use of antihypertensive medications.

^‡^Dyslipidemia was defined as total cholesterol 5.18 mmol/L; high-density lipoprotein cholesterol.

### SPECT MPI

MPI was performed in all patients using 2-day stress/rest protocol. Among the 86 study participants, 86 (100%) underwent rest MPI (37 positive results, 49 negative results) while only 70 (81.4%) participants underwent stress MPI (34 positive results, 36 negative results) because of poor physical condition or rejection of the remaining 16 patients. Stress MPI using adenosine stress test. Continuous intravenous injection through the cubital vein, connected by a tee, for a total of six minutes. At the 4th minute during the injection, the myocardial imaging agent 99mTc-MIBI 740MBq (20mCi) was injected through the tee. The electrocardiogram, blood pressure, heart rate and clinical symptoms of the patient were monitored throughout. Adenosine injection was stopped in the following cases:

- Significantly lower blood pressure;
- Angina pectoris;
- When dizziness, dyspnea, nausea, and profuse sweating are unbearable;
- Severe heart rhythm appears on the electrocardiogram or severe atrioventricular block and other abnormal degeneration.

30 minutes after the end of the injection of the imaging agent, lipid meal was performed, and MPI was performed after 90 minutes. The resting MPI was performed the next day.

The imaging apparatus was a GE Infinia Hawkeye 4 SPECT/CT with a parallel low-energy high-resolution collimator. Matrix 64 × 64, Magnification 1.0, gating acquisition. Image processing was performed with the ECT TOOLBOX software provided with SPECT/CT. Image analysis and interpretation of the results were done by two experienced nuclear medicine physicians. Myocardial perfusion was analyzed by employing the 17-segment model which was graded using a 5-point scoring system. The total score was calculated by summing the score for each segment [11].

### CDG acquisition

**Fig 1:**
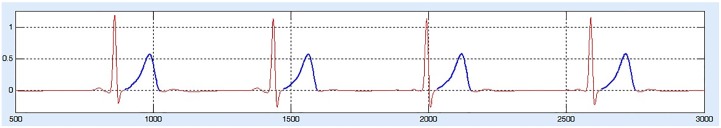
The *ST*-*T* segment of ECG used to construct CDG.

The routine 12-lead ECGs were recorded before rest MPI by using commercially available electrocardiograph (Mindray Bene-Heart R12, Shenzhen, China), with 1000-Hz sampling rate and 16-bit resolution. Before the recording, patients were requested to stay in the supine position for 20 seconds. All ECGs were analyzed by 1 independent experienced cardiologists blinded to patient characteristics. Of the 86 patients, 72 patients’ ECGs are non-diagnostic ECG and 14 patients’ ECGs are diagnostic ECG, where diagnostic ECG was defined as with persistent or transient horizontal or downsloping ST depression 0.05*mV* in 2 contiguous leads and/or *T* inversion 0.1*mV* in 2 contiguous leads with prominent *R* wave. The CDG of a patient was generated by extracting the cardiodynamics information from the *ST*-*T* loop of VCG [7], which was transformed from the *ST*-*T* segments of standard 12-lead ECG. First, the *ST*-*T* segment of the routine 12-lead ECG is identified and extracted first. Then, based on the fact that 12-lead ECG and 3-lead VCG can be linearly transformed into each other without loss of useful information content pertaining to the heart dynamics [12], we transform the *ST*-*T* segment of 12-lead ECG signal *x*(*t*) (Fig 1) into *ST*-*T* loop of VCG signal *V*(*t*). In fact, the CDG represents the rate of change in *V*(*t*), which can be described by the following three ordinary differential equations:

$$\begin{array}{c} \dot{V}\left(t\right)=F\left(V_{1}\left(t\right),V_{2}\left(t\right),V_{3}\left(t\right)\right) \end{array}$$ (1)

where *F*(*V*_1_(*t*), *V*_2_(*t*), *V*_3_(*t*)) = [*f*_1_(*V*(*t*)), *f*_3_(*V*(*t*), *f*_2_(*V*(*t*)] is an unknown nonlinear function vector, that is, the cardiodynamics information underlying the *ST*-*T* segment. Third, we employed an extension of the deterministic learning algorithm [13–15], to model the cardiodynamic *F*(*V*(*t*)), the following constant representation of *F*(*V*(*t*)) can be obtained:

$$\begin{array}{c} F\left(V\left(t\right)\right)=F^{NN}\left(\phi_{v}\right) \end{array}$$ (2)

where $F^{NN}\left(\phi_{v}\right)=\left[f_{1}^{NN}\left(\phi_{v}\right),f_{2}^{NN}\left(\phi_{v}\right),f_{3}^{NN}\left(\phi_{v}\right)\right]$ is the modeling results of the cardiodynamics, a constant representation of *F*(*V*(*t*)) is the trajectory of *ST*-*T* loop. Finally, CDG was obtained by plotting the cardiodynamics information *F*(*V*(*t*)) = *F*^*NN*^(*ϕ*_*v*_) in the three-dimensional *XYZ* coordinate system.

**Fig 2:**
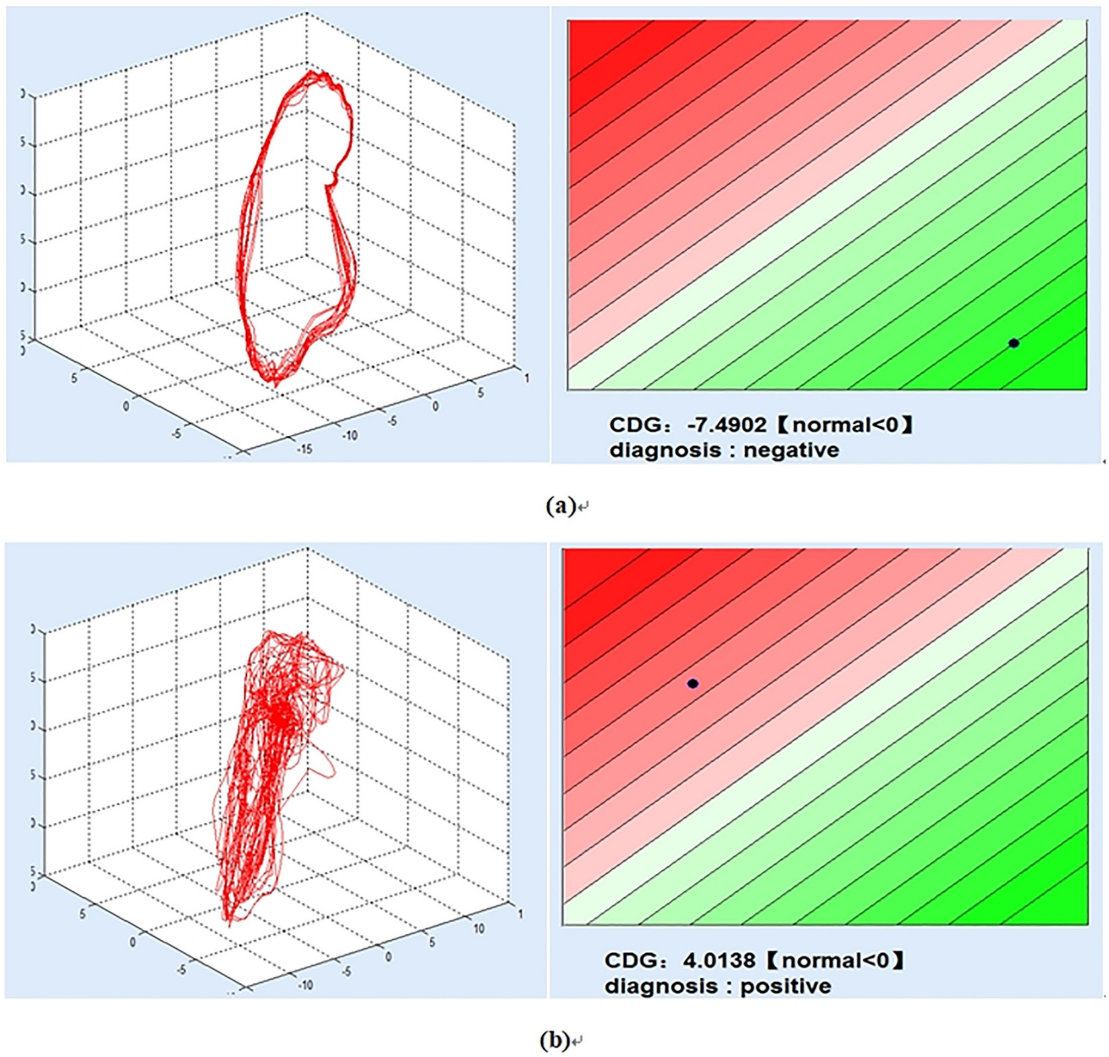
Two cases of CDG. (a): A case of a 23-year-old healthy subject. (b): A case of a 55-year-old female who suffered from ischemia.

By analyzing CDG morphology, it is found that the shapes of CDG remarkably differ between healthy controls (regular shapes, Fig 2(a)) and myocardial ischemia patients (irregular shapes, Fig 2(b)). It can be interrupted from the repolarization process of myocardial cell. Under normal conditions, repolarization is a homogeneous process. This results in a noticeably regular “annular” shape in CDG. Under myocardial ischemia, repolarization is a heterogeneous process. This results in an irregular, “scattered”, “nonannular” shape in CDG. These imply that significant correlations exist between CDG and myocardial ischemia [16].

Automated computer interpretation of CDG is achieved by evaluating spatial and temporal characteristics in CDG. Spatial heterogeneity index, based on the Lyapunov index [17] of CDG, is used to describe the spatial characteristic:

$$\begin{array}{c} SHI=\frac{1}{N}\sum_{n=1}^{N}ln\left(\frac{d_{n2}}{d_{n1}}\right) \end{array}$$ (3)

where *N* represents number of data points in CDG, *d*_*n*1_ represents the distance between the *n*-th data point and its nearest data point, and *d*_*n*2_ represents the distance between the *n*-th data point and its nearest data point after 10 steps. Temporal heterogeneity index, based on the Fourier transform [18] of the CDG, is used to describe the temporal characteristic:

$$\begin{array}{c} THI=argmin_{\lambda_{i}}\left|F\cdot e^{-0.1\lambda_{i}}\right| \end{array}$$ (4)

where *F* represents the Fourier transform of *X*′(*t*). Dispersion index (*DI*), based on *SHI* and *THI*, is used to describe the spatial-temporal dispersion of CDG morphology:

$$\begin{array}{c} DI=-0.0018\cdot THI-SHI+0.4 \end{array}$$ (5)

According to preliminary studies on volunteers [7, 8], a positive CDG graph is defined as *DI* > 0 and a negative CDG graph is defined as *DI* < 0.

### Statistic analysis

Different performance evaluation criteria were used to evaluate the predictive value of CDG in myocardial perfusion abnormalities. These are accuracy, sensitivity (SEN), specificity (SPE), positive predictive values (PPV), negative predictive values (NPV), positive likelihood ratio (LR+), negative likelihood ratio (LR-), Kappa value (*K*), which are calculated as follow:

$$\begin{array}{c} \begin{array}{cc} & Accuracy=\frac{TP+TN}{TP+FP+TN+FN}\times 100\%\\ & Sen=\frac{TP}{TP+FN}\times 100\%\\ & Spe=\frac{TN}{TN+FP}\times 100\%\\ & PPV=\frac{TP}{TP+FP}\times 100\%\\ & NPV=\frac{TN}{TN+FN}\times 100\%\\ & LR+=\frac{Sen}{1-Spe}\\ & LR-=\frac{1-Sen}{Spe}\\ & K=\frac{p_{0}-p_{e}}{1-p_{e}} \end{array} \end{array}$$ (6)

where TP, TN, FP and FN are true positives, true negatives, false positives and false negatives respectively, *p*_0_ = *Accuracy*, *p*_*e*_ = (*TP* + *FN*)(*TP* + *FP*) + (*TN* + *FP*)(*TN* + *FN*)/(*TP* + *FP* + *TN* + *FN*)^2^.

SEN is the percentage of actual positives that are correctly diagnosed. SPE is the percentage of actual negatives that are correctly diagnosed. PPV and NPA are the proportions of positive and negative results in statistics and diagnostic tests that are TP and TN results. Likelihood ratios are used for assessing the value of performing a diagnostic test. There exist two versions of the likelihood ratio, one for positive and one for negative test results, which are known as the LR+, and LR-. Kappa value is a measure of agreement between one specific medium and the gold standard and varies between 0 and 1.

In addition, receiver operating characteristic (ROC), a statistical tool that measures the ability of an index variable to diagnose either presence or absence of a characteristic, is also conducted for the performance evaluation in this study. Area under receiver operating characteristic (AUC) value, which is equal to the probability that a classifier will rank a randomly chosen positive instance higher than a randomly chosen negative one, is calculated for the analysis of the ROC curve.

## Results

In the clinic, patients always underwent both rest and stress MPI for diagnosis of coronary artery disease. Nevertheless, rest MPI can also be used alone to evaluate ischemia in certain situations such as patient’s poor physical condition, patient’s rejection, and positive results are mainly seen in patients with severe ischemia or myocardial infarction [19]. While, reverse distribution (in our study, there are 10 cases of reverse distribution) may be misdiagnosed as severe ischemia by rest MPI. Stress MPI is more sensitive for ischemia than rest MPI, but the negative result of stress MPI may lead to missed diagnosis of reverse distribution.

Thus, to more fully evaluate the predictive value of CDG in myocardial perfusion abnormalities. In this study, we conducted three experiments based on the SPECT MPI results:

1. In the first group, the rest SPECT MPI results of the 86 patients were compared with the CDG results, where the SPECT MPI ischemia score less than 2 was defined as normal (negative) otherwise was defined as myocardial ischemia (positive);
2. In the second group, the stress SPECT MPI results of the 70 patients were compared with the CDG results, the positive criterion of stress SPECT MPI was same with the first group;
3. In the third group, we compared the CDG results with the combination results of the rest and stress SPECT MPI results, if both rest and stress SPECT MPI results were negative, then we defined the combination result as negative, otherwise we defined the combination result as positive.

Three more cases (GB0527, GB0880 and GB0515) are given to show the effectiveness of CDG in assessing the myocardial perfusion abnormalities: two patients (GB0527 and GB0880) with normal ECG and exercise ECG, which may be missed diagnosed in clinical practice; one patient (GB0515) with abnormal ECG and exercise ECG, which often misclassified as myocardial ischemia.

**Fig 3:**
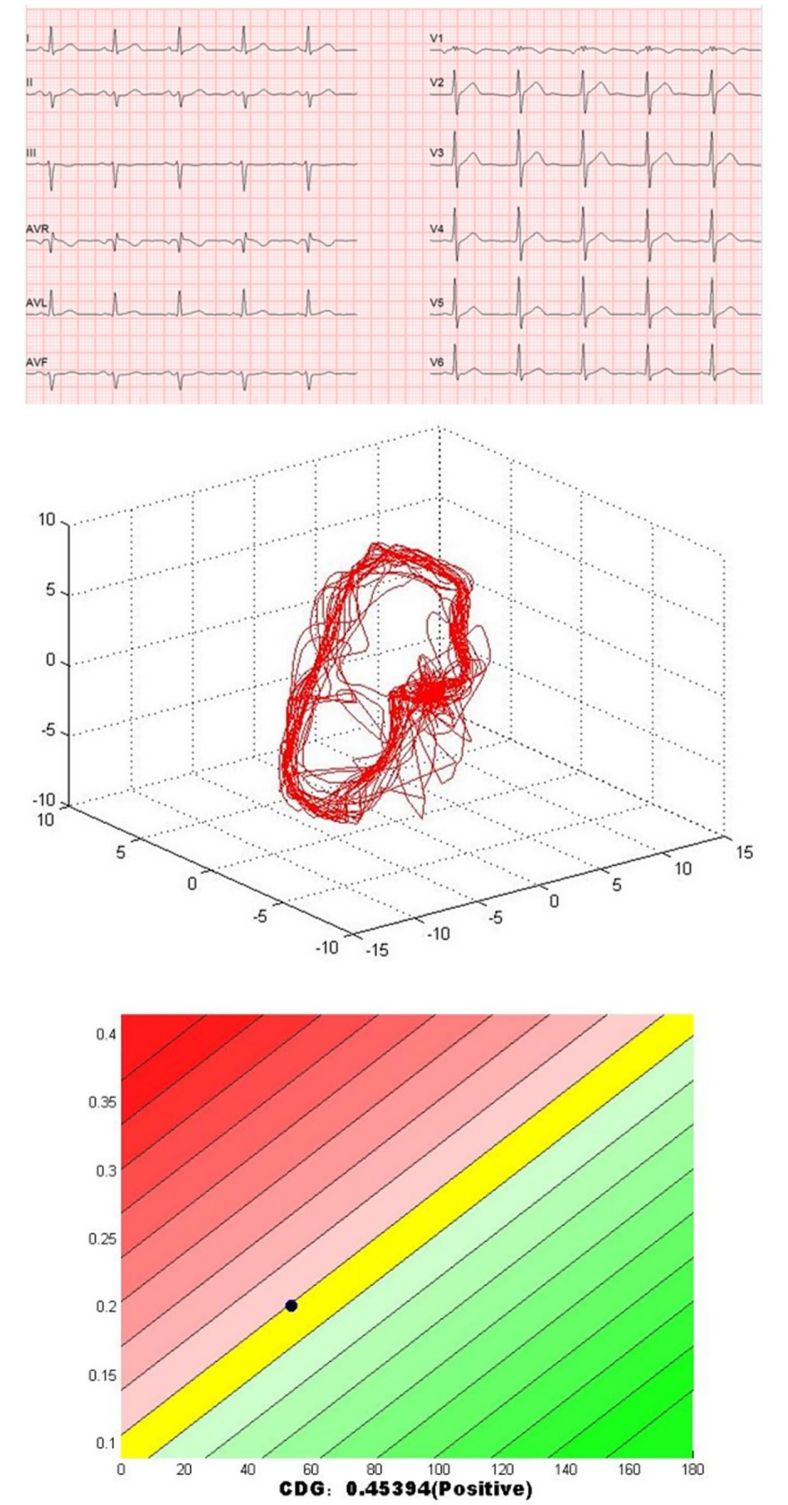
The ECG, CDG and *DI* of GB0527. GB0527 is a 50-year-old male patient, ECG is normal, and CDG is positive with *DI* = 0.45.

**Fig 4:**
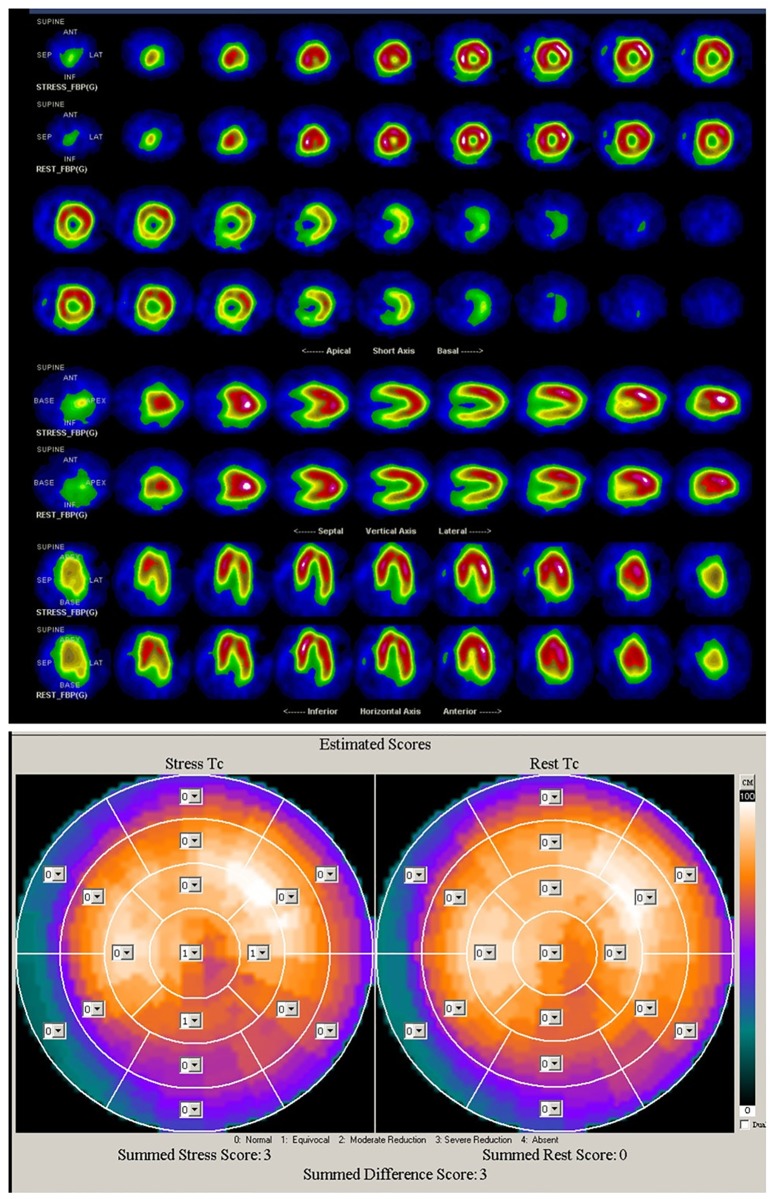
SPECT MPI results of GB0527. The rest and stress SPECT ischemia score of GB0527 are 0 and 3, respectively.

**Fig 5:**
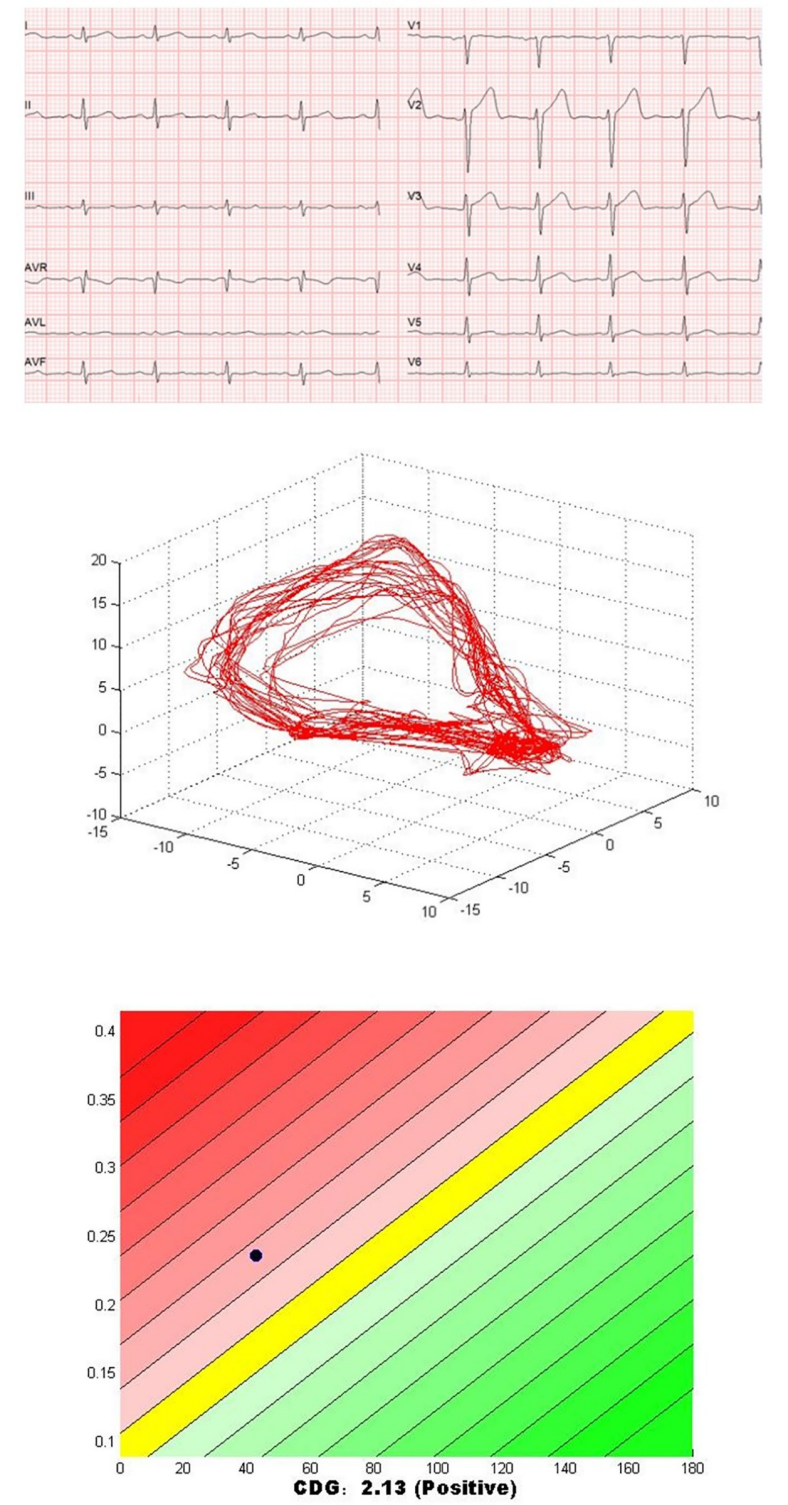
The ECG, CDG and *DI* of GB0880. GB0880 is a 42-year-old male patient with moderately severe stenosis, ECG is normal, and CDG is positive with *DI* = 2.13.

**Fig 6:**
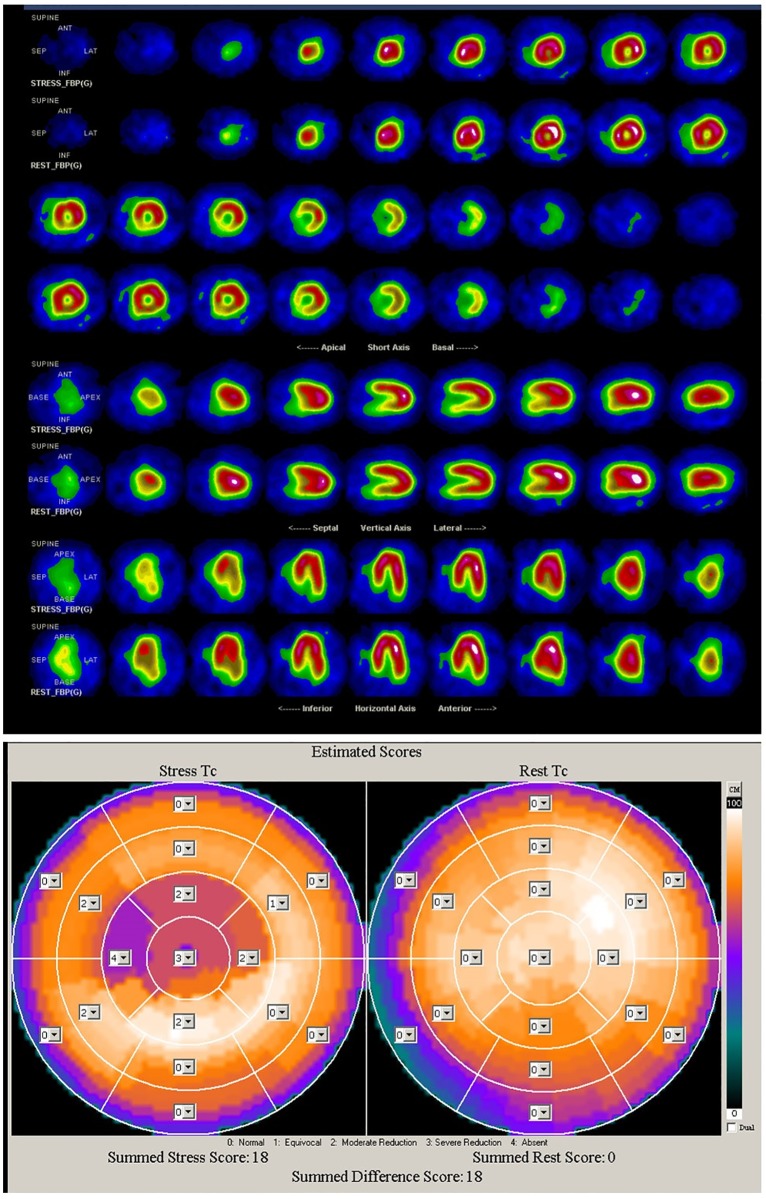
SPECT MPI results of GB0880. The rest and stress SPECT ischemia score of GB0527 are 0 and 18, respectively.

**Fig 7:**
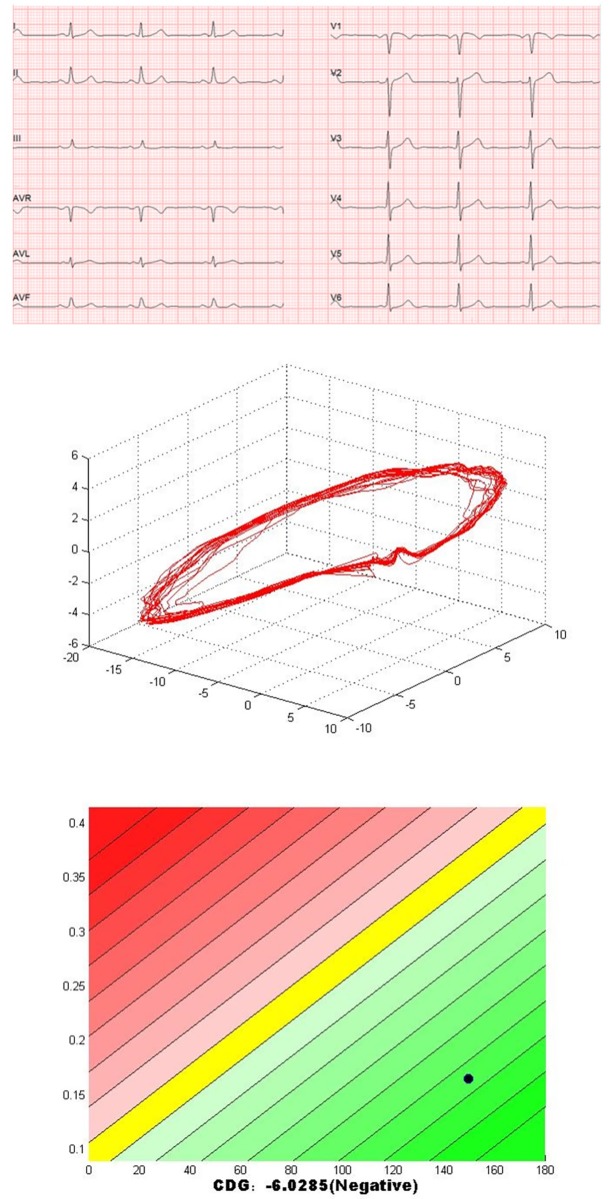
The ECG, CDG and *DI* of GB0515. GB0515 is a 68-year-old female patient was with the symptom of chest pain and abnormal ECG, and CDG is negative with *DI* = −6.03.

**Fig 8:**
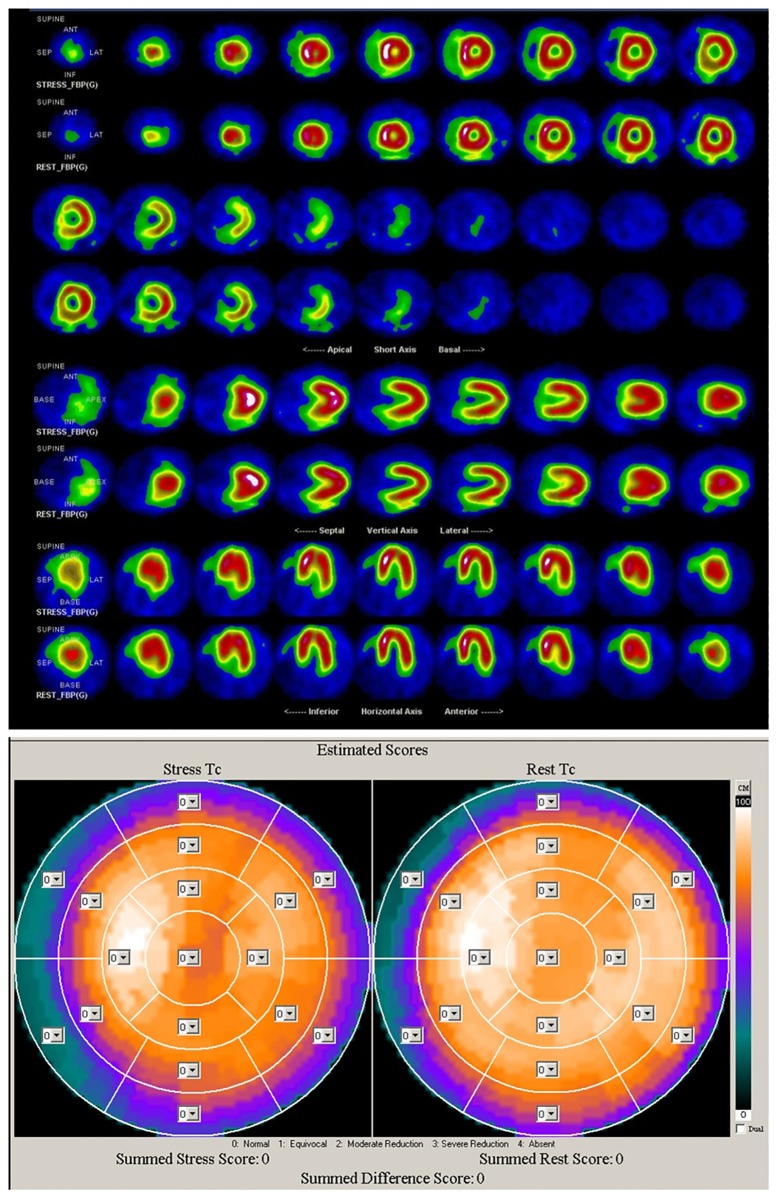
SPECT MPI results of GB0515. Both rest and stress SPECT ischemia score of GB0527 are 0.

The CDG result of GB0527 is shown in Fig 3. It can be seen that CDG morphology of GB0527 is a little scattered and *DI* = 0.45, which is consistent with the result of SPECT MPI as shown in Fig 4 (Summed Rest Score is 0, Summed Stress Score is 3). Figs 5 and 6 show the CDG result and SPECT MPI result of GB0880, a patient with moderately severe stenosis. It can be seen clearly the CDG result of GB0880 is positive with *DI* = 2.13. It is consistent with the result of SPECT MPI of GB0880 (Summed Rest Score is 0, Summed Stress Score is 18). In addition, it can be seen from Figs 3 and 5 that ECG signals of GB0527 and GB0880 are normal, which may be missed diagnosed in clinical practice and lead to fatal consequence. These indicate that CDG is more sensitive than conventional 12-lead ECG for ischemia detection. On the hand, GB0515 is a female patient with the symptom of chest pain and abnormal ECG as shown in Fig 7, thus GB0515 was first misclassified as myocardial ischemia. While, both the rest and stress SPECT MPI results are negative as shown in Fig 8 (Summed Rest Score is 0, Summed Stress Score is 0). This is consistent with CDG result shown in Fig 7. The CDG morphology of GB0515 is regular and *DI* = −6.03.

**Fig 9:**
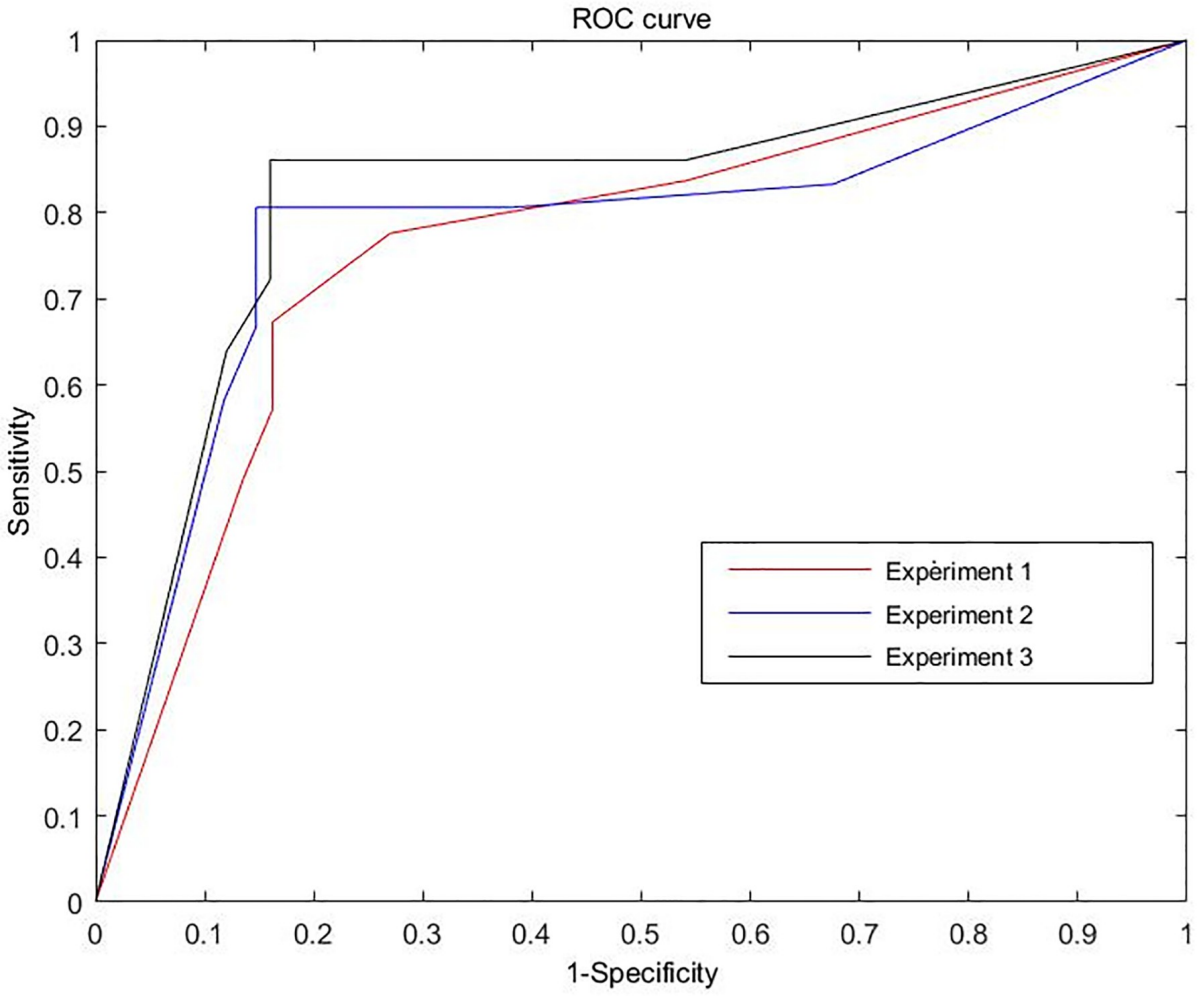
Receiver-operating characteristic curves of CDG for the three experiments. The areas under the three ROC curves were 0.759 (the 95% confidence interval 0.653 to 0.865), 0.756(the 95% confidence interval 0.632 to 0.881), and 0.799(the 95% confidence interval 0.691 to 0.907), respectively.

The results of the three experiments were shown in Tables 2 to 4. In the third experiment, CDG has an overall accuracy of 84.9%, sensitivity of 84.0%, specificity of 86.1%, positive predictive values = 89.4%, negative predictive values = 79.5%, positive likelihood ratio (LR+) = 6.04, and negative likelihood ratio(LR-) = 0.186. Generally, the accuracy of rest SPECT MPI for myocardial ischemia is lower than stress SPECT MPI, that is for a same patient, the ischemia score of rest SPECT MPI is usually lower than the stress SPECT MPI. While, there are also some special patients whose ischemia score of the rest SPECT MPI is higher than the stress SPECT MPI, this unusual phenomenon is named as reverse redistribution. Thus, we regard the third experiment as the final result to evaluate the clinical value of CDG in myocardial perfusion abnormalities. Using different critical CDG points, a receiver-operating characteristic curve was generated (Fig 9).

**Table 2:** Correlation between CDG and rest SPECT.

CDG	Rest SPECT MPI		Total
	positive(+)	negative(-)	
positive(+)	31(TP:83.8%)	16(FP:32.7%)	47
negative(-)	6(FN:16.2%)	33(TN:67.3%)	39
Total	37	49	86

Accuracy = 74.4%; Sensitivity = 83.8%; Specificity = 67.3%; Kappa value = 0.494; Positive predictive values (PPV) = 66.0%; Negative predictive values (NPV) = 84.6%; Positive likelihood ratio (LR+) = 2.56; Negative likelihood ratio (LR-) = 0.24.

**Table 3:** Correlation between CDG and stress SPECT.

CDG	Stress SPECT MPI		Total
	positive(+)	negative(-)	
positive(+)	29(TP:85.3%)	7(FP:19.4%)	36
negative(-)	5(FN:14.7%)	29(TN:80.6%)	34
Total	34	36	70

Accuracy = 82.9%; Sensitivity = 85.3%; Specificity = 80.6%; Kappa value = 0.658; Positive predictive values (PPV) = 80.6%; Negative predictive values (NPV) = 85.3%; Positive likelihood ratio (LR+) = 4.40; Negative likelihood ratio (LR-) = 0.17.

**Table 4:** Correlation between CDG and SPECT.

CDG	SPECT MPI		Total
	positive(+)	negative(-)	
positive(+)	42(TP:84%)	5(FP:13.9%)	47
negative(-)	8(FN:16%)	31(TN:86.1%)	39
Total	50	36	86

Accuracy = 84.9%; Sensitivity = 84%; Specificity = 86.1%; Kappa value = 0.693; Positive predictive values (PPV) = 89.4%; Negative predictive values (NPV) = 79.5%; Positive likelihood ratio (LR+) = 6.04; Negative likelihood ratio (LR-) = 0.186.

Moreover, we also conducted a statistical analysis of the CDG results of the 10 patients with reverse redistribution. Reverse redistribution was defined as either normal exercise perfusion and defective redistribution or an exercise defect worsened at redistribution [20]. It is relatively common among patients with coronary artery spasm (CAS) [21] or patients with recent myocardial infarction [22]. Of the 10 cases of reverse redistribution, 9 patients’ CDG are positive which can be preliminarily inferred that the reverse redistribution has high consistency with CDG. In the following study, we will further study the consistency between the CDG and the reverse redistribution and its clinical significance.

**Remark 1**: Arrhythmia is a common heart disease in middle-aged and elderly people, and has attracted extensive attention from researchers and scholars. In the paper, arrhythmia patients were not included mainly due to the following reasons: (i) the study aims to evaluate the clinical utility of CDG in predicting the myocardial perfusion abnormalities rather than arrhythmia; (ii) the CDG of arrythmia patients has not been studied intensively currently. Some preliminary studies on CDG of arrhythmia patients have been done and show that CDG morphology is very disorderly. Further research is underway to reveal the correlation between the CDG morphology and arrhythmia.

## Discussion

CDG demonstrates myocardial perfusion abnormalities in a novel manner using a 3-dimensional graphic representation which is based on the routine 12-lead ECG signals. This is the first clinical research of CDG for assessment of myocardial perfusion abnormalities. The results demonstrate that CDG has a high value for the detection of myocardial perfusion abnormalities. With the standard of invasive SPECT MPI, CDG yields a total accuracy of 84.9%, a sensitivity of 84.0%, and a specificity of 89.4%.

In fact, CDG reflects the cardiac electrical conduction, that is, CDG is negative for normal conduction, and otherwise it will be positive. While, besides myocardial ischemia there are other factors can change the stability of cardiac electrical conduction, such as myocardial injury arrhythmia, Purkinje fiber injury. Myocardial ischemia is a more common reason for the abnormal of cardiac electrical. It is the main reason for the false positive cases of CDG in detection of myocardial perfusion abnormalities. In addition, ECG noise is another reason for false positive.

For some patients with local old myocardial infarction or ischemia diseases persisted for a long time, the instability of cardiac electrical conduction will be turned into stability because of the myocardial remodeling, in these cases, the ischemia exists while the CDG is negative. That is the main reason for the false negative cases of CDG in detection of myocardial perfusion abnormalities. In the study, 3 of the 8 false negative patients have a history of myocardial infarction and 2 of them had undergone stent surgery. Related research to further improve the performance of CDG in assessment of myocardial perfusion abnormalities is underway, better performance can be expected.

In addition, CDG is the 3-dimensional graphics of cardiac dynamics information, the rate of change in VCG. Obviously, CDG is fundamentally different from VCG. VCG reflects the repolarization vector while CDG is the rate of change in VCG. So, CDG is more sensitive than VCG for the detection of myocardial perfusion abnormalities. Moreover, lots of studies have proved the potential of VCG in principle for ischemia diagnosis, while VCG has not been established as a typical method, probably because it is difficult to interpret [23]. As CDG is generated by routine 12-lead ECG signals and can be interpreted directly by an objective indicator, obviously CDG is also more convenient than VCG.

**Fig 10:**
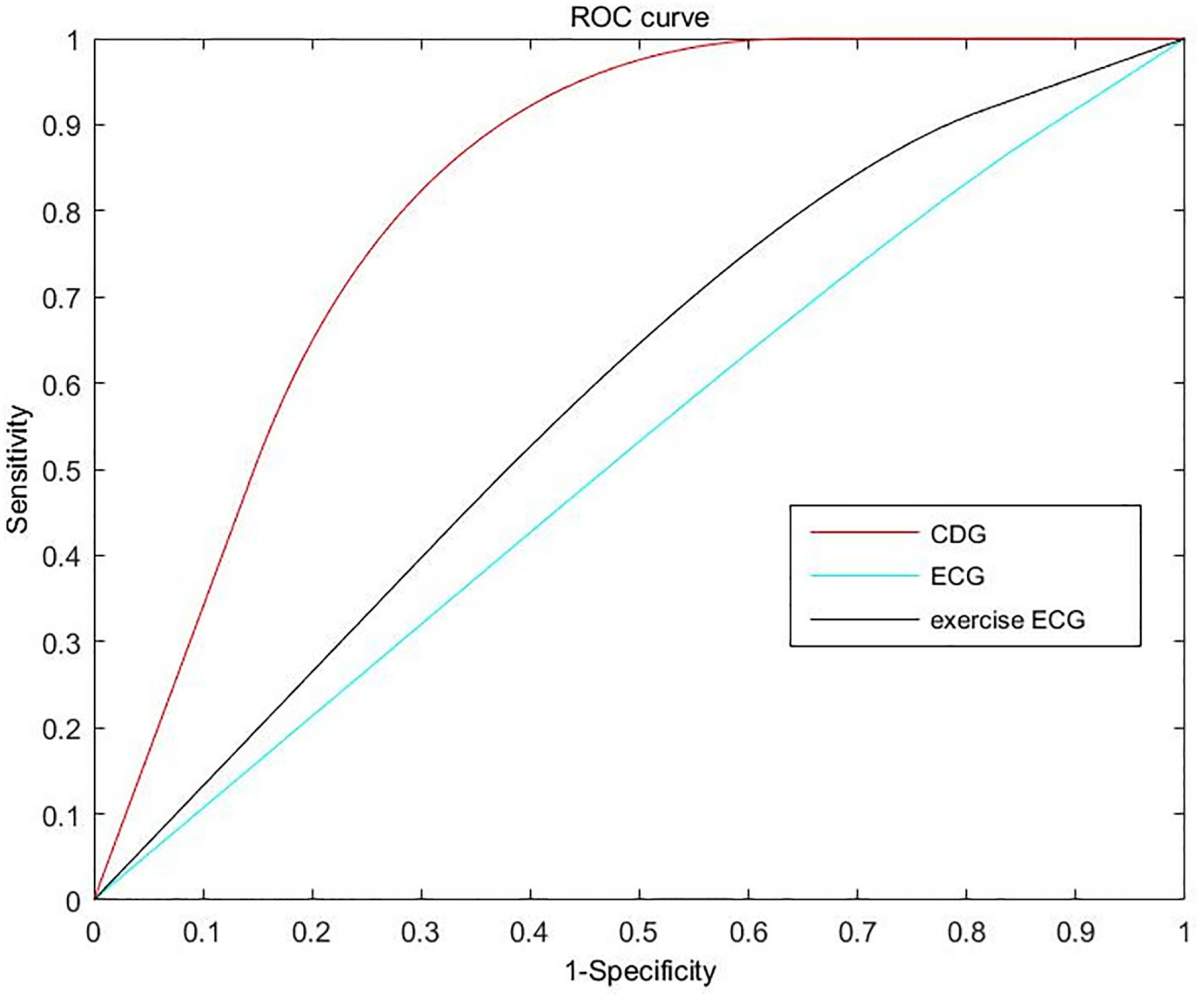
Receiver-operating characteristic curves of CDG, ECG and exercise ECG. The areas under the three ROC curves were 0.759 (the 95% confidence interval 0.653 to 0.865), and 0.521(the 95% confidence interval 0.396 to 0.645), 0.621(the 95% confidence interval 0.497 to 0.744), respectively.

To further show the clinical values of CDG versus routine ECG and exercise ECG, receiver-operating characteristic curves of CDG, ECG and exercise ECG (all of the 86 enrolled patients performed exercise ECG before several days of SPECT MPI) are shown in Fig 10. It can be seen clearly that CDG is more valuable than ECG and exercise ECG, at the same time, CDG is as easy as ECG. To sum up, CDG is a noninvasive, convenient, and useful tool for assessing the myocardial perfusion abnormalities. It is expected to be a cost-effective method for initial assessment of ischemic heart diseases.

## Limitation

First, our findings are based on a single-center study with limited patients and clinical examination. Of the 86 patients, only 29 (34%) patients underwent coronary angiography, and 17 (20%) patients underwent coronary CTA, where coronary angiography and coronary CTA are the most important tools in the clinic for CAD detection. Thus, the findings may not be directly generalized to other populations. Multicenter studies are required to further evaluate the predictive value of CDG in myocardial perfusion abnormalities detection in larger patients with more detailed clinic information including the results of coronary angiography, coronary CTA, the final hospital diagnosis and so on. In addition, our study was not powered to address the physiological mechanism of CDG and the correlation between CDG and myocardial perfusion abnormalities. Further studies (including virtual heart simulation experiments, animal experiments, more clinical trials etc.) are needed to study CDG’s physiological mechanism. Second, there are other heart diseases influencing the results of SPECT MPI, such as myocarditis, cardiomyopathy, coronary spasm, myocardial bridge, are not discussed in detail. The effect of these heart diseases on the results of SPECT MPI should be discussed intensively in detail in the further studies.

## Supporting information

S1 File The minimal data set. (ZIP)
